# Assessment of Physical Activity During Chemotherapy and/or Immunotherapy for Non-Small Cell Lung Cancer (NSCLC): Protocol of the APACHIE-01 Study

**DOI:** 10.3390/clinpract15080139

**Published:** 2025-07-25

**Authors:** Dirk Rades, Laura Doehring, Christian Staackmann, Maria Karolin Streubel, Stefan Janssen, Tobias Bartscht, Sabine Bohnet

**Affiliations:** 1 Department of Radiation Oncology, University of Lübeck, 23562 Lübeck, Germany; stefan.janssen@uksh.de; 2 Department of Radiation Oncology, University Medical Center Schleswig-Holstein, Lübeck Campus, 23538 Lübeck, Germany; laura.doehring@uksh.de (L.D.); christian.staackmann@uksh.de (C.S.); mariakarolin.streubel@uksh.de (M.K.S.); 3 Medical Practice for Radiotherapy and Radiation Oncology, 30161 Hannover, Germany; 4 Department of Hematology, Oncology and Stem Cell Transplantation, Helios Hospital Schwerin, 19055 Schwerin, Germany; tobias.bartscht@helios-gesundheit.de; 5 Department of Pulmonology, University Medical Center Schleswig-Holstein, Lübeck Campus, 23538 Lübeck, Germany; sabine.bohnet@uksh.de

**Keywords:** chemotherapy, immunotherapy, non-small cell lung cancer, physical activity, smart phone

## Abstract

Background/Objectives: Most patients with non-small cell lung cancer (NSCLC) receive chemo- and/or immunotherapy, which can be associated with adverse events including fatigue. Affected patients may not be able to receive the complete chemo- and/or immunotherapy as planned. In this context, patients may benefit from maintaining their physical activity, which can be challenging. An app reminding patients to perform a certain number of steps may have a positive effect on physical activity during chemo- and/or immunotherapy. Such an app is under development and will be tested in a prospective trial. The current APACHIE-01 study (NCT06993896) is required for proper sample size calculation and design of the planned trial. Methods: The main goal of the APACHIE-01 study is to evaluate patterns and predictors of physical activity during chemo- and/or immunotherapy for locally advanced or metastatic NSCLC. The primary endpoint is the assessment of the mean number of steps per week during the first three cycles of chemo- and/or immunotherapy for lung cancer. The baseline value is represented by the mean number of steps during the last week prior to chemotherapy and/or immunotherapy. Secondary endpoints include associations between mean number of steps per week and a pain score, a distress score, and a fatigue score. The recruitment of the required 38 patients should be completed within 4 months and the treatment period will be 9–10 weeks (three cycles of chemo- and/or immunotherapy), resulting in a total running time of approximately 6 months. The APACHIE-01 study will contribute to the optimal design of a subsequent prospective trial.

## 1. Introduction

Lung cancer belongs to the most common types of solid cancer in Europe and Northern America [1]. Most patients with non-small cell lung cancer (NSCLC) receive chemo- and/or immunotherapy [2]. Many patients with distant metastases (stage IV disease) are treated with chemo- and/or immunotherapy alone or combined with palliative radiotherapy. Moreover, a considerable number of patients assigned to thoracic surgery receive neoadjuvant chemo- and/or immunotherapy without irradiation. The chemo- and/or immunotherapy regimens used for NSCLC can be associated with adverse events including fatigue [3,4,5,6,7,8,9,10,11,12]. Patients who experience significant treatment-related toxicity may not be able to receive the complete chemo- and/or immunotherapy as planned. Physical activity may be helpful in this context. In a retrospective study of 184 cancer patients receiving neoadjuvant chemotherapy, including 31 patients with NSCLC, adherence to an exercise program was associated with fewer dose reductions and delays of chemotherapy [13]. Moreover, several studies performed in patients with lung cancer suggested that physical activity had a positive effect on the patient’s quality of life [12,14,15,16,17,18,19,20]. In a retrospective study of 50 patients who received chemotherapy for advanced or recurrent lung cancer, low physical activity was negatively associated with survival [21]. Thus, physical activity prior to and during chemo- and/or immunotherapy for lung cancer appears important.

Maintaining physical activity during a course of chemo- and/or immunotherapy may be challenging for the patients, particularly if they experience treatment-related fatigue or other adverse events [22,23]. In a randomized pilot study of 40 patients with low physical activity who were receiving or had already received chemo- and/or immunotherapy for advanced stage NSCLC, patients of the intervention group received a FitBit^®^ Flex 2 accelerometer, a 15-minute in-person teaching session, and twice-daily gain-framed text messages [24]. Physical activity was measured with a questionnaire evaluating weekly minutes of moderate and vigorous exercise at baseline and after 12 weeks. In addition, steps were counted in the intervention group at baseline and after 6 and 12 weeks with the accelerometer. The intervention led to a more pronounced increase in physical activity when compared to usual care (control group). In the intervention group, the step count after 12 weeks has not significantly decreased when compared to baseline [24]. The question arose whether an easy-to-use mobile app installed on the patient’s smart phone reminding patients several times daily to perform a certain number of steps will also have a positive effect on their physical activity during chemo- and/or immunotherapy. The patient’s smart phone will also be used for counting the steps. Such an app is currently under development within the Interreg project Health Advancing Technologies for Elderly (HeAT). When it becomes available, the app will be tested in a prospective phase 2 trial. For proper sample size calculation and design of the trial, the current prospective study is required, which evaluates the weeks with a mean number of steps per week at baseline and during chemo- and/or immunotherapy for locally advanced or metastatic NSCLC.

## 2. Experimental Design and Materials

### 2.1. Objectives and Endpoints

The main goal of the APACHIE-01 study is to evaluate patterns and predictors of physical activity during chemo- and/or immunotherapy for lung cancer. The primary endpoint is to assess the mean number of steps per week during the first three cycles of chemo- and/or immunotherapy for lung cancer. The baseline value is represented by the mean number of steps during the last week prior to chemotherapy and/or immunotherapy. Any type of smart phone is allowed, as long as it has a step counter. The steps can be directly obtained from an iPhone^®^ that has an integrated pedometer. For other types of smart phones, the patients are asked to use the app Pedometer^®^ by Google Play^®^ (https://play.google.com/store/apps/details?id=com.tayu.tau.pedometer&hl=de (accessed on 18 July 2025)) to reduce the risk of bias. In case of difficulties with installing the app, the patient receives support from a health care professional involved in the APACHIE-01 study. The steps will be directly assessed from the patient’s smart phone by a health care professional to avoid unnecessary data transfer, which is in line with the local data protection regulations. The patients will receive instructions by a health care professional involved in this study for how to carry the smart phone while walking (i.e., in a trouser pocket) to ensure consistency of the measurements.

In addition, the following secondary endpoints will be evaluated:

1. Associations between mean number of steps per week and a pain score (prior to and weekly during the first three cycles of chemotherapy and/or immunotherapy);
2. Associations between mean number of steps per week and a distress score (prior to and weekly during the first three cycles of chemotherapy and/or immunotherapy);
3. Associations between mean number of steps per week and a fatigue score (prior to and weekly during the first three cycles of chemotherapy and/or immunotherapy).

### 2.2. General Trial Design and Duration

The APACHIE-01 study is a monocentric prospective study that aims to obtain information about the physical activity during chemo- and/or immunotherapy for lung cancer. The recruitment of all 38 patients should be completed within 4 months. The treatment period will be 9–10 weeks (three cycles of chemo- and/or immunotherapy). This equals a total running time for the trial of approximately 6 months.

### 2.3. Eligibility Criteria

Criteria for inclusion and exclusion in the APACHIE-01 study are shown in Table 1.

### 2.4. Sample Size Calculation

The main goal of this trial is to describe the pattern of physical activity in patients with NSCLC during chemo- and/or immunotherapy, to evaluate potential predictors of those patterns and to assess potential correlations with patient reported outcomes (pain, distress, and fatigue scores). To reach this goal, this trial will be the first in which the mean number of steps per week performed at baseline and during each week of chemo- and or immunotherapy will be collected in a standardized prospective way. The trial is exploratory in nature, with the aim to generate scientific hypotheses to be investigated in future clinical trials. Due to the exploratory character of this trial, sample size estimation is not based on a specific statistical hypothesis system. Instead, the sample size is derived by the following simple statistical reasoning and feasibility aspects.

The goal of this study is to compare mean number of steps per week between various study weeks. Therefore, the effect size is considered as a relevant parametrization of the shift in mean values. The effect size is defined as the mean of the paired difference divided by its standard deviation and, therefore, constitutes a standardized scale indicating the number of standard deviations by which the means differ. Assuming that an effect size of 0.5 for the mean number of steps per week between two study weeks is clinically interesting/relevant and worth detecting, 34 patients are required to achieve a power of 80% with a level of significance of 5% (two-sided) using a *t*-test for comparing two paired means. This sample size is also considered feasible in this single-center trial setting, considering the planned study duration. Assuming that 10% of the patients may not be included in the descriptive analyses due to lack of data, a total number of 38 patients should be enrolled to this exploratory, hypothesis-generating trial.

## 3. Detailed Procedure

### 3.1. Assessments

The following parameters will be recorded prior to the start of chemo- and/or immunotherapy: medical history, concomitant diseases, concomitant medication, physical examination, demographics (age, gender), body mass index (BMI), Karnofsky performance score, tumor histology, tumor stage, metastatic sites, history of smoking, type of planned chemo- and/or immunotherapy, mean number of steps (during the last week).

The following parameters will be assessed during the course of the trial:

1. Mean number of steps: The mean number of steps per week will be obtained weekly during the first three cycles of chemo- and/or immunotherapy (nine weeks) from the patient’s smart phone.
2. Pain score: Pain will be assessed prior to chemo- and/or immunotherapy, weekly during the first three cycles of chemo- and/or immunotherapy, and at end of treatment with a numeric rating scale (self-assessment, ranging from 0 points = no pain to 10 points = maximum pain). Furthermore, the intake of analgesics will be documented.
3. Distress score: Distress will be assessed prior to chemo- and/or immunotherapy, weekly during the first three cycles of chemo- and/or immunotherapy, and at end of treatment with the National Comprehensive Cancer Network (NCCN) Distress Thermometer (self-assessment, ranging from 0 points = no distress to 10 points = maximum distress) [25,26].
4. Fatigue score: Fatigue will be assessed prior to chemo- and/or immunotherapy, weekly during the first three cycles of chemo- and/or immunotherapy, and at end of treatment with the Fatigue Assessment Scale (FAS) (self-assessment, ranging from 0 points = no fatigue to 50 points = maximum fatigue) [©FAS (Fatigue Assessment Scale): ild care foundation (www.ildcare.nl (accessed on 18 July 2025))].
5. Adverse Events: Adverse events will be assessed on an ongoing basis according to Common Terminology Criteria for Adverse Events (CTCAE) version 5.0 [27].

The corresponding timeline is shown in Table 2.

### 3.2. Systemic Treatment

#### 3.2.1. Chemotherapy

Patients with non-squamous carcinoma will receive carboplatin Area Under the Curve (AUC) 5 plus pemetrexed 500 mg/m^2^. Patients with squamous carcinoma will receive Carboplatin AUC 5 plus paclitaxel 175 mg/m^2^ in a 3-week cycle for up to four cycles of therapy according to local standards.

#### 3.2.2. Possible Acute Side Effects of Chemotherapy

Acute side effects of chemotherapy include, but are not limited to, mucositis, nausea, vomiting, fatigue, pain, diarrhea, alopecia, decreased leucocyte count, decreased neutrophil count, anemia, and thrombocytopenia. Monitoring will be performed according to local standard procedures, which include weekly control of complete blood count and clinical chemistry.

#### 3.2.3. Immunotherapy

Patients will receive either pembrolizumab 200 mg every three weeks before chemotherapy, followed by a maintenance phase with immunotherapy only (or durvalumab 1500 mg + tremelimumab 1 mg/kg every three weeks before chemotherapy followed by a maintenance phase with immunotherapy only).

#### 3.2.4. Possible Acute Side Effects of Immunotherapy

Acute and subacute side effects of immunotherapy include, but are not limited to, rash, colitis, pneumonitis, nephritis, and thyroiditis. All patients will be monitored for side effects according to local standard procedures, which include weekly lab controls and physical examination on day 1 of each cycle.

### 3.3. Statistical Methods

#### 3.3.1. General Considerations

All data recorded in the case report forms describing the study population (demographic and clinical characteristics recorded at baseline), physical activity, safety, and patient-reported outcomes will be analyzed descriptively. Stratified two-by-two contingency tables will be analyzed using Cochran–Mantel–Haenszel tests. Comparison of ordinal variables between treatment arms will be performed using the asymptotic Wilcoxon–Mann–Whitney test, replaced by its exact version in case of ordinal categories with small numbers of categories and/or sparse data within categories. Any shift in location of quantitative variables between study groups will be performed with the Wilcoxon–Mann–Whitney tests as well. Mean number of steps per week between groups of patients will be compared by means of *t*-tests for independent groups. To compare paired data between study visits, *t*-tests for paired observations will be applied. All patients who have started the chemotherapy and/or immunotherapy and provide data on the primary endpoint will be analyzed in this exploratory clinical trial.

#### 3.3.2. Primary Endpoint

The primary aim of the study is to describe the pattern of physical activity during three chemotherapy cycles. Physical activity will be operationalized by means of the mean number of steps per week, resulting in a total of three measurements per treatment cycle in all patients with any documented activity data during treatment cycles. According to our knowledge and experience, compliance of patients is expected to be high and the rate of missing data is supposed to be low. Moreover, the primary endpoint is based on the derivation of one specific value for each patient from several scores obtained over a comparably short period of time. Considering the scientific question addressed in the APACHIE-01 study, missing values will not be imputed regardless of any intercurrent events. For calculating the mean number of steps per week, days on which no steps are recorded will be excluded and considered as missing.

To describe the individual trajectories of mean numbers of steps over the course of the three chemotherapy and/or immunotherapy cycles, spaghetti plots will be created. In addition, summary statistics by week nested within chemotherapy cycles will be calculated using the number of subjects with non-missing data, mean, standard deviation, median, first quartile, third quartile, minimum, and maximum. The absolute change from baseline will be summarized using the same statistics. This analysis also allows us to assess whether the mean numbers of steps per week are primarily changing within a therapy cycle or whether a differential effect throughout the 3 cycles can be observed. To further differentiate the patterns within the therapy cycles from those between therapy cycles, the mean steps per week will be further aggregated: For the former analysis, the arithmetic means of the mean number of steps over the therapy cycles are calculated for each week. For the latter analysis, the arithmetic means over the weeks are calculated for each therapy cycle.

The above-mentioned analyses will also be performed stratified by potential prognostic baseline characteristics and treatment variables.

The changes over time in mean numbers of steps per week will be further assessed using a restricted maximum likelihood-based mixed model for repeated measures (MMRM) under the assumption of missing at random (MAR). The dependent variable of this model will be the mean numbers of steps measured in week 1 and week 3 of each of the three treatment cycles. The mean numbers of steps at the End-of-Study-Visit will not be included in this analysis, as the EOT time may be different for each subject and the respective mean number of steps may likely be confounded by the reason for the treatment discontinuation. The model will include the fixed categorical effects of week (1, 2, 3), treatment cycle (1, 2, 3), and week-by-cycle interaction, the fixed continuous covariate of mean baseline number of steps, as well as those baseline characteristics that show a trend towards heterogeneous mean numbers of baseline steps as expressed by a *p*-value less than 15% in the univariate analyses.

The random subject effects are not explicitly formulated in the model form but will be modeled as part of the within-subject error correlation structure. For this purpose, an unstructured covariance matrix will be used. If the analysis using the unstructured covariance matrix fails to converge, additional covariance structures such as heterogeneous compound symmetry and compound symmetry will be tested. In such cases, the covariance structure converging to the best fist, as determined by Akaike’s information criterion, will be used as the primary analysis. The Kenward–Roger approximation will be used to estimate denominator degrees of freedom.

The least square means resulting from the model above will be evaluated and plotted for each week. If sample sizes are sufficiently large, subgroups defined by baseline covariates may be investigated further to explore the mean number of steps over time for patients within each subgroup. Due to the nature of this study, any findings from these analyses will also be considered exploratory.

#### 3.3.3. Secondary Endpoints

First of all, the pain score, the distress score, and the fatigue score will be subjected to descriptive statistical analysis in analogy to the methods described in Section 3.2.2. The correlations between mean numbers of steps per week and the specific pain, distress, and fatigue scores will be assessed by means of Spearman rank correlation coefficients stratified by week nested within chemotherapy cycles. Any resulting correlation coefficient will only be considered as a hint towards statistical significance if the resulting *p*-value is less than 0.01 as a criterion for adjusting for multiple comparisons. To further differentiate the patterns within each therapy cycle from those between therapy cycles, the number of steps and the scores of the patient-reported outcomes will be further aggregated and subjected to the above-mentioned correlation analysis.

### 3.4. Data Protection, Data Management, and Distribution of Results

Data protection, data management, and distribution of results will be performed according to our institutional standard procedures that have been described in previous study protocols [28,29].

## 4. Expected Results and Discussion

Patients treated with systemic therapy, i.e., chemo- and/or immunotherapy, for NSCLC can experience significant fatigue leading to a decline in physical activity. As a consequence, the patients may not be able to receive the complete planned systemic therapy, which has a negative impact on the patient’s prognosis. This study investigates the patients’ physical activity during three cycles of chemo- and/or immunotherapy, represented by the mean number of steps per week. It is expected that the mean number of steps will decrease during the study period of 9–10 weeks. The results of the APACHIE-01 study are mandatory for the proper design of a subsequent prospective trial testing the impact of a reminder app on the patients’ physical activity.

However, when using the data of the APACHIE-01 study, its limitations need to be considered, namely the single-center nature, the limited sample size, and the possibility of incorrect step counting in case of problems regarding the sensor sensitivity or if a patient does not carry the smart phone as advised. Moreover, the results of the APACHIE-01 study may not be applicable to patients with stage I, stage II, or stage IIIA disease.

Physical activity may improve the compliance with the protocols of chemo- and/or immunotherapy. The retrospective study of Wonders et al. investigated the benefit of exercise training during adjuvant or neoadjuvant chemotherapy in 184 patients with an advanced malignant disease [13]. The most common type of malignancy was breast cancer (35.9%), followed by NSCLC (16.8%) and Hodgkin’s lymphoma (11.4%). The patients underwent at least 12 weeks of personalized training. Patients with an exercise adherence of more than 84.3% experienced fewer delays and reductions of their chemotherapy doses. Although not shown in that study, receiving the chemotherapy as planned in terms of dose and timing will likely have a positive impact on a patient’s prognosis. This assumption may be supported by another retrospective study including 50 lung cancer patients treated with chemotherapy for advanced or recurrent disease [21]. In that study, low physical activity was found to be negatively associated with survival (hazard ratio = 4.35; *p* = 0.029). These results suggest that maintaining physical activity during a course of chemo- and/or immunotherapy is important for the outcomes of patients with NSCLC.

However, maintaining the pre-treatment level of physical activity can be a challenge for patients suffering from fatigue or other treatment-associated acute toxicities [22,23]. These patients may benefit from support by an app reminding them of physical activity or walking a certain number of steps each day. A previous pilot study from the United States investigated patients with low physical activity who received or were currently under chemo- and/or immunotherapy for advanced-stage NSCLC [24]. The intervention, which included a FitBit^®^ Flex 2 accelerometer, a 15-minute in-person teaching session, and twice-daily gain-framed text messages, increased the patients’ physical activity. After 12 weeks, no significant decrease in the step count compared to baseline was noticed. The idea of our group within the Interreg project HeAT is to develop a mobile app that is installed on the patient’s smart phone and reminds them several times per day of performing a minimum number of steps. The benefit of this mobile app is planned to be investigated in a prospective trial (APACHIE-02), for which the results of the present study (APACHIE-01) are required. The app that will be tested in the APACHIE-02 trial is not yet available but already under development.

## Figures and Tables

**Table 1:** Inclusion and exclusion criteria for the APACHIE-01 study.

Inclusion criteria	Histologically proven locally advanced (stage IIIB or IIIC) or metastatic (stage IV) non-small cell lung cancer Indication for chemotherapy and/or immunotherapy Possession of and ability to use a smart phone that includes a step counter Willingness to wear the smart phone close to the body at any time Age ≥ 18 years Written informed consent Capacity of the patient to consent
Exclusion criteria	Small-cell lung cancer Karnofsky performance score < 60 Thoracic surgery within 3 months prior to chemotherapy and/or immunotherapy Expected non-compliance

**Table 2:** Timeline of enrolment, interventions, and assessments.

	Prior to Chemo- and/or Immunotherapy	Weekly During Chemo- and/or Immunotherapy	End of Study
**Demographics**	X		
**Medical history and concomitant diseases**	X		
**Concomitant medication**	X	X	X
**Physical examination**	X		
**Karnofsky performance score**	X	X	X
**Planned chemo- and/or immunotherapy**	X		
**Planned radiotherapy**	X		
**Treatment given as planned?**		X	X

**Study-related procedures**			
**Informed consent**	X		
**Inclusion criteria**	X		
**Exclusion criteria**	X		
**Assessment of adverse events**	X	X	X
**Mean number of steps per week**	X	X	X
**Pain score**	X	X	X
**Distress score**	X	X	X
**Fatigue score**	X	X	X

## Data Availability

Further information regarding this trial is available at clinicaltrials.gov (identifier: NCT06993896).

## Abbreviations

The following abbreviations are used in this article:

AUC	Area Under the Curve
BMI	Body Mass Index
CTCAE	Common Terminology Criteria for Adverse Events
FAS	Fatigue Assessment Scale
NCCN	National Comprehensive Cancer Network
NSCLC	Non-Small Cell Lung Cancer
